# The Association Between Post-stroke Depression, Aphasia, and Physical Independence in Stroke Patients at 3-Month Follow-Up

**DOI:** 10.3389/fpsyt.2018.00374

**Published:** 2018-08-20

**Authors:** Shuo Wang, Chun-Xue Wang, Ning Zhang, Yu-Tao Xiang, Yang Yang, Yu-Zhi Shi, Yi-Ming Deng, Mei-Fang Zhu, Fei Liu, Ping Yu, Gabor S. Ungvari, Chee H. Ng

**Affiliations:** ^1^Department of Neurology, Beijing Tian Tan Hospital, Capital Medical University, Beijing, China; ^2^Department of Neuropsychiatry and Behavioral Neurology and Clinical Psychology, Beijing Tian Tan Hospital, Capital Medical University, Beijing, China; ^3^China National Clinical Research Center for Neurological Diseases, Beijing, China; ^4^Center of Stroke, Beijing Institute for Brain Disorders, Beijing, China; ^5^Beijing Key Laboratory of Translational Medicine for Cerebrovascular Disease, Beijing, China; ^6^Department of Clinical Psychology, Capital Medical University, Beijing, China; ^7^Unit of Psychiatry, Faculty of Health Sciences, University of Macau, Taipa, Macau; ^8^Department of Interventional Neuroradiology, Beijing Tian Tan Hospital, Capital Medical University, Beijing, China; ^9^University of Notre Dame Australia, Marian Centre and Graylands Hospital, Perth, WA, Australia; ^10^Department of Psychiatry, University of Melbourne, Melbourne, VIC, Australia

**Keywords:** aphasia, ischemic stroke, depression, PSD, physical independence

## Abstract

**Objective:** Few studies have examined the association between post-stroke depression (PSD), aphasia, and physical independence in Chinese patients. This study investigated the above association in stroke patients in China at 3-month follow-up.

**Methods:** Altogether 270 patients within 14 days after ischemic stroke were recruited and followed up at 3 months. PSD, aphasia, and physical functional status were measured using the Stroke Aphasia Depression Questionnaire (SADQ), Western Aphasia Battery (WAB), and modified Rankin Scale (mRS), respectively. Patients with mRS total score >2 were considered as having “physical dependence.”

**Results:** Out of 248 patients at 3-month follow up, 119 (48%) were rated as having physical dependence. Multiple logistic regression analyses revealed that female (*p* = 0.04; OR = 2.2; 95% CI: 1.0–5.1), more severe stroke at admission (*p* < 0.01; OR = 1.4; 95% CI: 1.3–1.5), and more severe PSD at 3 months (*p* = 0.01; OR = 1.05; 95% CI: 1.01–1.1) were independently associated with physical dependence at 3 months.

**Conclusions:** Greater PSD and stroke severity were independently associated with physical dependence at 3 months after stroke. Aphasia was also associated with physical dependence but the relationship was not significant. Early and effective depression screening, treatment and stroke rehabilitation appear to be important to improve the physical outcome and reduce the burden of stroke survivors.

## Introduction

Post-stroke depression (PSD) is one of the most common psychiatric comorbidities in stroke survivors, with a prevalence ranging from 20 to 65% (1–4). PSD is significantly associated with poor treatment adherence and increased risk of disability, mortality, stroke recurrence, and poor quality of life (5, 6). Aphasia occurs in about a third of ischemic stroke patients and is associated with impaired activity of daily life (7, 8) and higher risk of PSD (9, 10). Poor functional outcome after stroke could result in significant personal distress and family burden (11). Understanding the association between PSD, aphasia and physical independence is hence important to develop comprehensive treatment strategies for stroke survivors.

In China, the lifetime prevalence of stroke was 2.08% (95% CI, 2.02–2.13%) (12) in 2017, which translates to ~2.9 million stroke patients. An Italy study found PSD was the only significant factor related to functional recovery from discharge to 3-month follow-up after stroke but not aphasia (13). To the best of our knowledge, there are no studies that have investigated the independent association between PSD, aphasia, and physical independence in stroke survivors in China. This study thus aimed to examine the association between PSD, aphasia, and physical independence in Chinese stroke patients at 3-month follow-up.

## Methods

### Participants and study setting

This prospective cohort study was conducted between April 2014 and October 2015 in the Stroke Centre of Beijing Tiantan Hospital. A total of 320 patients were consecutively screened if they fulfilled the following criteria: (1) aged 18 years or older; (2) had an acute ischemic stroke within 14 days according to the WHO diagnostic criteria (14) confirmed by computed tomography (CT) or magnetic resonance imaging (MRI); and (3) had the ability to provide informed consent and complete the assessment. Exclusion criteria included: (1) history of language impairment; (2) drug and alcohol abuse and severe psychiatric disorders; (3) other major medical conditions, such as Parkinson's disease; and (4) severe cognitive deficit defined by the Mini Mental State Examination (MMSE) total score <18 (15, 16). The first three were excluded based on the self-reported pre-stroke histories by unstructured interviews and the last was made at the start of the study. The study protocol was approved by the ethics committee of Beijing Tiantan Hospital, Capital Medical University. All participants provided written informed consent.

### Measurement instruments and evaluation

Assessment was conducted at baseline and 3 months after index ischemic stroke. Patients' socio-demographic and clinical characteristics at baseline were recorded via a review of electronic medical records and confirmed by a clinical interview conducted by trained research neurologists. Severity and type of stroke was assessed with the National Institutes of Health Stroke Scale (NIHSS) (17–19) and the Trial of Org 10172 Acute Stroke Treatment (TOAST) (20, 21).

Physical independence and degree of handicap was evaluated using the modified Ranking Scale (mRS) at 3 months, with mRS total score >2 indicating physical dependence (22, 23). As most depression scales cannot be used in stroke patients with aphasia due to their impaired communication ability, the severity of depressive symptoms at 14 ± 2 days and 3 months after index stroke was measured using the 21-item Stroke Aphasic Depression Questionnaire (SADQ) (24, 25) with total score ranges from 0 to 63, ≥19 indicating the presence of PSD, ≥ 22 indicating moderate depressive symptoms and ≥ 26 indicating major depressive symptoms. The SADQ relies on external observation of emotional behavior by nursing staff and family members in recent time. The hospital version (SADQ-H) focus on the recent week. The severity and type of aphasia was evaluated with the Aphasia Quotient (AQ) derived from the Western Aphasia Battery (WAB), with AQ < 93.8 indicating the presence of aphasia (26). The language assessment was also conducted at 3-month follow-up with much missing data on AQ score, so the data was not analyzed.

### Statistical analysis

Data were analyzed with SPSS 21.0 (SPSS Inc., Chicago, IL, USA). Socio-demographic and clinical variables were compared between physical independence and dependence groups using Chi-square test, independent sample *t*-test and Mann-Whitney *U*-test, as appropriate. Independent correlates of the physical dependence at 3 months were examined using multivariate logistic regression analysis with the “enter” method. The outcome at 3 months was the dependent variable, while variables that significantly differed between both groups in the univariate analyses were entered as independent variables. Significance was set at 0.05 (two-tailed).

## Results

Out of 320 patients with ischemic stroke who were consecutively screened, 270 fulfilled the study entry criteria and participated in the study, giving a participation rate of 84.4%. At baseline, 160 (59.3%) patients had aphasic symptom with the incidence of PSD was 47.5% compared with 29.1% in non-aphasiac patients (*p* < 0.01). At the 3-month assessment, 22 patients dropped out due to lack of interest, moving house or other unknown reasons. Of the 248 patients who completed the 3-month assessment, 119 (48.0%) had physical dependence.

Table 1 shows the socio-demographic and clinical characteristics of the whole sample and separately by outcome. Patients with physical dependence were less likely to be married, and have large-artery atherosclerosis (LAA) TOAST type and pulmonary infection, but more likely to be female, have aphasia at baseline and more severe depressive symptoms at both baseline and 3 months after stroke. In addition, they had higher NIHSS scores at baseline, and higher SADQ scores at baseline and 3 months (all *p*-values < 0.05).

**Table 1:** Comparison of demographic and clinical variables between physical independence and dependence groups.

	**Total sample (*****n*** = **248)**		**Physical independence (*****n*** = **129)**		**Physical dependence (*****n*** = **119)**		**Statistics**		
	***N***	**%**	***N***	**%**	***N***	**%**	**χ^2^**	**df**	***p***
Female	72	29.0	26	20.2	46	38.7	10.2	1	**0.001**
Higher education (high middle school or above)	88	35.5	49	38.0	39	32.8	0.7	1	0.39
Being married	229	92.3	124	96.1	105	88.2	5.4	1	**0.02**
Living with others	235	94.8	123	95.3	112	94.1	0.1	1	0.66
Diabetes	61	24.6	29	22.5	32	26.9	0.6	1	0.42
Hypertention	156	62.9	87	67.4	69	58.0	2.3	1	0.12
Hyperlipidemia	33	13.5	19	14.7	14	11.8	0.4	1	0.49
Cardiac disease	21	8.5	10	7.8	11	9.2	0.1	1	0.67
Stroke history	58	23.4	27	20.9	31	26.1	0.9	1	0.34
TOAST type (LAA)	209	84.3	115	89.1	94	79.0	4.8	1	**0.02**
Aphasia at baseline	148	59.7	66	51.2	82	68.9	8.1	1	**0.004**
Depressive symptoms at baseline	100	40.3	39	30.2	61	51.3	11.3	1	**0.001**
Moderate/Major depressive symptoms at baseline	54	21.8	15	11.6	39	32.8	16.2	1	**<0.001**
Depressive symptoms at 3 months	85	34.7	26	20.2	59	50.9	25.4	1	**<0.001**
Moderate/Major depressive symptoms at 3 months	48	19.6	8	6.2	40	34.5	31.0	1	**<0.001**
Pulmonary infection at baseline	36	14.5	13	10.1	23	9.3	4.2	1	**0.03**
Upper gastrointestinal hemorrhage at baseline	8	3.2	3	2.3	5	4.4	0.2	1	0.63
Cardiac diseases at baseline	12	4.8	5	3.9	7	5.9	0.5	1	0.46
Urinary infection at baseline	11	4.4	3	2.3	8	6.7	2.8	1	0.09
Deep venous thrombosis at baseline	20	8.1	5	3.9	15	12.6	6.3	1	**0.01**
	**Mean**	***SD***	**Mean**	***SD***	**Mean**	***SD***	**T/Z**	**df**	***p***
Age (years)	57.3	14.2	57.3	14.0	57.3	14.6	0.01	246	0.9
Stroke duration from onset to admission	6.1	6.1	6.3	6.1	5.9	6.0	−1.2	__a	0.22
NIHSS at baseline	8.3	5.3	5.1	3.7	11.8	4.5	−10.1	__a	**<0.001**
SADQ-H at baseline	14.5	7.9	12.5	7.2	16.8	8.0	−4.2	__a	**<0.001**
SADQ at 3 months	12.8	8.0	9.7	7.1	16.2	7.6	−6.3	__a	**<0.001**

*Values p < 0.05 are bolded; a, Mann-Whitney U-test; LAA, Large-Artery Atherosclerosis; NIHSS, National Institutes of Health Stroke Scale; SADQ-H, Stroke Aphasic Depression Questionnaire (Hospital version); SADQ, Stroke Aphasic Depression Questionnaire; TOAST, Trial of Org 10172 in Acute Stroke Treatment*.

The independent correlates of physical dependence are shown in Tables 2, 3. Due to collinearity between SADQ-H (Hospital version using during hospitalization) and SADQ score, two multivariate logistic regression analyses were performed with SADQ-H and SADQ separately. Finally, female, NIHSS score at admission and SADQ score at 3 months were independently associated with physical dependence at 3-month follow up (adjusted *R*^2^ = 0.410 in Table 2 and adjusted *R*^2^ = 0.423 in Table 3).

**Table 2:** Independent correlates of physical dependence at 3 months^#^.

	**Ischemic stroke patients**		
**Variables**	***P***	**OR**	**95%CI**
Feale	**0.04**	2.2	1.0,5.1
Being married	0.22	0.3	0.08,1.7
TOAST type (LAA)	0.75	1.1	0.4,3.5
Aphasia at baseline	0.32	1.4	0.6,2.9
SADQ-H score at baseline	0.72	1.08	0.9,1.05
Pulmonary infection at baseline	0.74	1.1	0.4,3.4
Deep venous thrombosis at baseline	0.27	2.4	0.5,11.5
NIHSS at admission	**<0.01**	1.4	1.3,1.5

*Values p < 0.05 are bolded; a, Mann-Whitney U-test; LAA, Large-Artery Atherosclerosis; NIHSS, National Institutes of Health Stroke Scale; SADQ-H, Stroke Aphasic Depression Questionnaire (Hospital version); TOAST, Trial of Org 10172 in Acute Stroke Treatment*.

# *The severity of depressive symptoms was tested by SADQ-H at baseline and SADQ at 3 months. There was collinearity between SADQ-H at baseline and SADQ at 3 months, therefore they were not entered in the analysis concurrently*.

**Table 3:** Independent correlates of physical dependence at 3 months^#^.

	**Ischemic stroke patients**		
**Variables**	***P***	**OR**	**95%CI**
Female	0.08	2.0	0.9,4.8
Being married	0.26	0.4	0.08,1.9
TOAST type (LAA)	0.80	1.1	0.3,3.4
Aphasia at baseline	0.53	1.2	0.6,2.5
SADQ score at 3 months	**0.01**	1.05	1.01,1.1
Pulmonary infection at baseline	0.69	1.2	0.4,3.6
Deep venous thrombosis at baseline	0.30	2.2	0.4,10.3
NIHSS at admission	**<0.01**	1.4	1.2,1.5

*Values p < 0.05 are bolded; a, Mann-Whitney U test; LAA, Large-Artery Atherosclerosis; NIHSS, National Institutes of Health Stroke Scale; SADQ, Stroke Aphasic Depression Questionnaire; TOAST, Trial of Org 10172 in Acute Stroke Treatment*.

# *The severity of depressive symptoms was tested by SADQ-H at baseline and SADQ at 3 months. There was collinearity between SADQ-H at baseline and SADQ at 3 months, therefore they were not entered in the analysis concurrently*.

## Discussion

Generally about a third of patients with ischemic stroke have poor functional outcome at different follow-up time points (27, 28). In this study, the prevalence of physical dependence was 48% using the mRS cut-off score, which is similar to the 5-year prevalence of poor functional outcome (45%) in another study with the same measure (29). However, any direct comparison should be done with caution due to the different assessment measures, follow-up time points, and demographic characteristics.

A report of the American Heart Association/American Stroke Association in 2017 and meta-analyses found that approximately a third of stroke patients develop PSD at any point after stroke (30–32). A large-scale prospective cohort study in China involving over 2,000 stroke patients found that the cumulative incidence of PSD at 1 year after the index stroke was 42% (4). Depression was found predictive of worse functional outcome in an updated meta-analysis about the impact of depression on stroke outcome (33). While up to 62–70% in aphasic patients with stroke were diagnosed as major depression according to DSM-III-R criteria at 3 months and 1 year after stroke (9, 10, 34) which was higher than our results. The reason may due to the different scales, population and time points. Aphasia have been considered for the potential association with PSD with inconsistent conclusion (35, 36). We found that the incidence of PSD in stroke patients with aphasia and non-aphasia was significantly different indicating aphasia may be a risk factor of the development of PSD.

In this study, the incidence of PSD was 34.7% at 3 months after stroke, which was independently associated with physical dependence. Similar findings were also reported previously (37–39). Depressive symptoms after stroke could result in behavioral and biological abnormalities, such as poor treatment adherence and dysregulation in autonomic system activation, which in turn, could lead to physical dependence (11, 37). However, since more than half of the patients suffered from aphasia in this sample, the use of the SADQ required the input of nursing staff and/or family members. Therefore, we could not exclude the possibility that nursing staff and family members were unable to distinguish between insomnia, irritability, poor appetite, anxiety, and depressive symptoms, which would cause bias in the incidence of PSD to an uncertain extent.

In this study, aphasia was measured using the AQ that covered complete aphasia, motor aphasia, sensory aphasia, transcortical mixed aphasia, transcortical motor aphasia, transcortical sensory aphasia, anomic aphasia, and conduction aphasia. Aphasia may lead to communication or comprehension difficulties, social avoidance and decreased attention, which is associated with physical dependence in stroke survivors (40). However, this finding was only confirmed in the univariate, but not in the multivariate analyses in this study. It is speculated that the association between aphasia and poor physical independence was moderated by other variables, such as depressive symptoms and severity of stroke. In addition, traditional Confucian culture favors family support and inter-dependence, particularly for family members with illness. For example, 94.8% of patients in this study were living with others. Thus, the strong family support may have offset the association between aphasia and physical dependence.

The association between demographic characteristics and physical dependence in stroke survivors have been inconsistent (41, 42). In this study, only female was independently associated with physical dependence, which is supported by previous findings (43, 44). The gender difference in physical independence in stroke survivors may be related to menstrual cycles, neuro-endocrine regulation and more frequent physical comorbidities, such as diabetes, atrial fibrillation, and coronary heart disease in women with stroke (45). As expected, stroke severity as measured by the NIHSS was positively associated with physical dependence, which is consistent with previous findings (27, 29).

There are several methodological limitations to this study. First, this was a single-center study with relatively small sample size, therefore the findings could not be generalized to all stroke patients in China. Second, depressive symptoms were measured using the SADQ based on the observation by nursing staff or family members. There may be a gap between observer-rated and self-reported measures of depression, although there are a number of self-reported measures specific for aphasia such as the Visual Analogue Mood Scales (VAMS) (46), Visual Analogue Self-Esteem Scales (VASES) (47), Disc Intensity Scale Circles (DISCS) (48), and Dynamic Visual Analogue Mood Scales (D-VAMS) (49). We chose SADQ from the perspective of relatively short items, easy operation and short time. Third, some important variables related to physical dependence, such as the use of medication, treatment adherence, the size and lesions of infarcts and the missing data about aphasia at 3-month, were not evaluated in the 3 months assessment.

In conclusion, physical dependence at 3-month follow up was common in Chinese stroke patients, which was associated with gender, greater PSD and stroke severity. Aphasia was also associated with physical dependence but the relationship was not significant. Our findings call for early and effective depression screening, treatment, and stroke rehabilitation to improve physical outcome and reduce the burden of stroke survivors in China.

## Author contributions

SW and C-XW: study design. SW, NZ, YY, Y-ZS, Y-MD, M-FZ, FL, PY, and Y-TX: collection, analysis and interpretation of data. SW, C-XW, and Y-TX: drafting of the manuscript. GU, CN, and Y-TX: critical revision of the manuscript. All coauthors approval of the final version for publication.

### Conflict of interest statement

The authors declare that the research was conducted in the absence of any commercial or financial relationships that could be construed as a potential conflict of interest.
